# Melioidosis of the Head and Neck: A Case Series from Eastern India

**DOI:** 10.3390/idr12030011

**Published:** 2020-10-29

**Authors:** Srujana Mohanty, Saurav Sarkar, Baijayantimala Mishra

**Affiliations:** 1 Department of Microbiology, All India Institute of Medical Sciences, Bhubaneswar, Odisha 751019, India; bm_mishra@hotmail.com; 2 Department of ENT, All India Institute of Medical Sciences, Bhubaneswar, Odisha 751019, India; ent_saurav@aiimsbhubaneswar.edu.in

**Keywords:** *Burkholderia pseudomallei*, melioidosis, neck abscess

## Abstract

Melioidosis is an emerging entity in India. Though it is a potentially fatal disease, prognosis is excellent with early detection and appropriate management, especially of localized infections like abscesses of the head and neck area. We report nine cases of focal abscesses in the head and neck region due to *Burkholderia pseudomallei*, the causative agent of melioidosis, presenting to our hospital within a span of two-and-half years. Since melioidotic abscesses in the cervicofacial and head and neck region are likely to be confused with cold abscesses caused by *Mycobacterium tuberculosis* in a tuberculosis-endemic country like India, increased vigilance is necessary because of the widely divergent treatment modalities of the two disease entities.

## 1. Introduction

Melioidosis, a potentially fatal disease caused by the saprophytic Gram-negative soil and water bacterium *Burkholderia pseudomallei*, is endemic, widespread and of immense public health importance in subtropical areas such as Southeast Asia and northern Australia [1,2]. In recent times, it is being increasingly reported from beyond its endemic boundaries and is considered as an emerging and evolving disease in several other regions of the globe such as China, Sri Lanka, North and South America and India [1,2,3].

The clinical spectrum of *B. pseudomallei* infection can be extremely varied, ranging from localized infection to fulminant septic shock with severe systemic manifestations [1,3]. Pneumonia is the most common clinical presentation accounting for over half the cases followed by genitourinary infection, skin infection and deep-seated organ abscesses [2,3]. All age groups can develop melioidosis, but incidence peaks between the ages of 40 and 60 years with approximately 75–81% of cases presenting during the rainy season [2,3,4]. The disease is more common in the rural areas and predominantly affects people in regular contact with soil and water e.g., rice farmers [2,4]. Common risk factors considered important in the development of melioidosis include diabetes mellitus, alcoholism, chronic renal disease, chronic liver disease, malignancy, connective tissue diseases, and immunosuppressive treatments, including steroids [1,3,4]. Untreated, the disease has a mortality rate reaching as high as 95% and a rate of 50% even after antibiotic therapy [3,4]. The usual antibiotic regimen consists of administration of prolonged parenteral ceftazidime as intensive therapy followed by oral trimethoprim-sulfamethoxazole as maintenance therapy.

Melioidosis has been reported as an uncommon cause of neck abscess except in cases of paediatric melioidosis in Thailand and Malaysia, where suppurative parotitis and other forms of head and neck involvement account for up to 40% of cases of melioidosis in children [4,5,6]. Apart from this, only scattered case reports of melioidosis of the head and neck region from different geographical areas of the world are available in literature, including India [7,8,9,10,11,12]. Melioidotic abscess in the head and neck can be a diagnostic challenge and needs to be differentiated from other common causes of head and neck abscess such as *Staphylococcus aureus*, *Streptococcus* species, atypical mycobacteria and *Mycobacterium tuberculosis* in view of the widely different therapeutic regimens. We report a series of nine cases of melioidosis with head and neck involvement, presenting to a tertiary care referral centre outlining their clinical and bacteriological summaries (Table 1). Our aim is to increase the awareness for this particular clinical entity of *B. pseudomallei* infection to enable correct identification of the etiologic agent and timely institution of appropriate therapy.

### 1.1. Case 1

A 49-year-old woman presented to the hospital with a 20-day history of an enlarging painful mass on the left side of neck, fever, poor appetite, and weight loss of 5–6 kg since the start of her symptoms. She was a housewife from a semi-urban area and a known case of diabetes mellitus on medication, but with poorly controlled glycaemic status. She had no history of contact with tuberculosis. On examination, the neck mass was found to be located on the left upper third cervical region abutting left of the mandible, measuring approximately 5.5 × 4 cm, firm, and tender with local rise of temperature and overlying erythema. Vital signs were stable with a pulse rate of 98/min, regular respiratory rate of 22/min, blood pressure of 100/74 mm Hg, and temperature of 39.2 °C. She weighed 44 kg. Laboratory studies showed leucocytosis (total leucocyte count 13.9 × 10^9^/L; 80% polymorphs, 16% lymphocytes, 2% eosinophils, 2% monocytes), mild thromobocytosis (platelet count 475 × 10^9^/L), raised erythrocyte sedimentation rate (ESR) 60 mm/h, and low hemoglobin 101 g/L. Blood metabolic panel suggested poor diabetes control (random blood sugar 351 mg/dL, fasting blood glucose 202 mg/dL, postprandial blood sugar 382 mg/dL), with urea 28 mg/dL and creatinine 1mg/dL.

**Figure 1:**
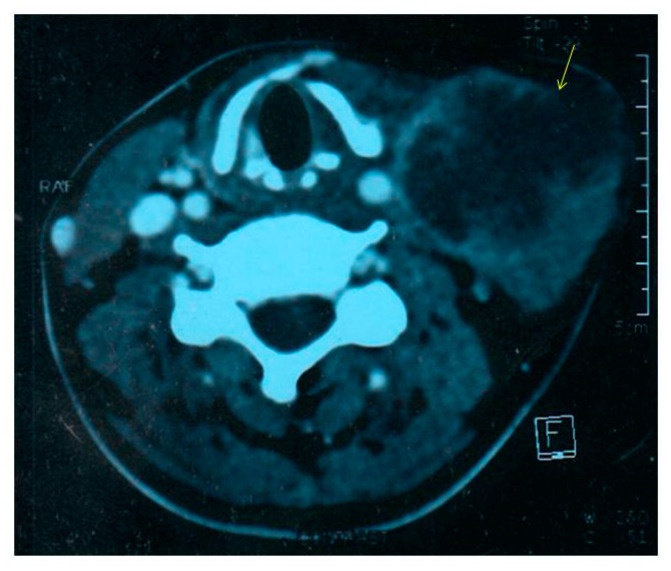
Contrast-enhanced CT neck showing left cervical abscess.

**Figure 2:**
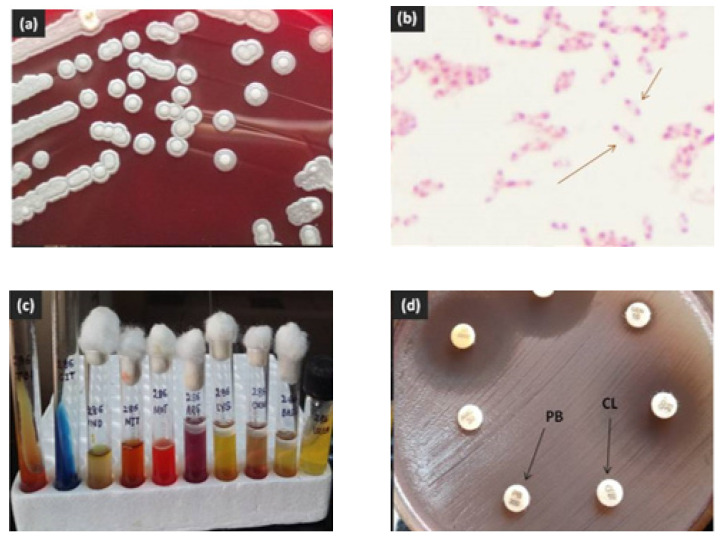
Identification of *B. pseudomallei* by (**a**) growth on blood agar, (**b**) “safety pin” appearance on Gram stain, (**c**) biochemical reactions, and (**d**) resistance to polymyxin B 300U (PB) and colistin 10 µg (CL) disks.

Contrast enhanced computed tomography (CECT) scan of the neck showed the presence of a large, heterogenous enhancing hypodense nodal mass in left cervical region (Figure 1), likely to represent a pyogenic or tuberculous abscess. A fine needle aspirate of the pus sample from the mass showed sheets of neutrophils, degenerated cells, and lymphocytes in a dirty background with no granulomas, necrosis or atypical cells. Mycobacterial smear and culture were negative. Bacterial culture yielded an oxidase-positive gram-negative rod, with an unusual pattern of antibiotic resistance with resistance to colistin, polymyxin B and aminoglycosides. Blood culture was negative. The pus isolate was identified as *Burkholderia pseudomallei* by its characteristic growth on blood agar and MacConkey’s agar (small, smooth, nonlactose fermenting colonies after overnight incubation, which turned pink with a metallic sheen after 48 h and dry and wrinkled after 96 h of incubation), typical morphology on microscopy (motile, gram negative bacilli with bipolar staining giving a “safety-pin” appearance) and standard biochemical tests (Figure 2) [10,11,12]. The isolate was sensitive to ceftazidime, co-trimoxazole, imipenem, meropenem, doxycycline and amoxicillin—clavulanate [13]. Minimum inhibitory concentration (MIC) of ceftazidime was 4 µg/mL by Etest (bioMérieux, Marcy l’Etoile, France).

Following the culture report, the patient underwent incision and drainage of the abscess mass and was commenced on intravenous (IV) ceftazidime administered as intensive therapy (2 gm 8-h) for a prolonged duration of three weeks followed by oral cotrimoxazole (Trimethoprim 240 mg and Sulfamethoxazole 1200 mg) twice daily as maintenance therapy. Glycemic control was optimized with insulin therapy. The fever subsided within a week of ceftazidime therapy followed by symptomatic healing of the neck lesion. The patient was discharged from the hospital on the 25th day of admission with advice to continue cotrimoxazole for six months. On follow-up two-and-a-half months later, complete healing of the lesion was noted.

### 1.2. Case 2

**Figure 3:**
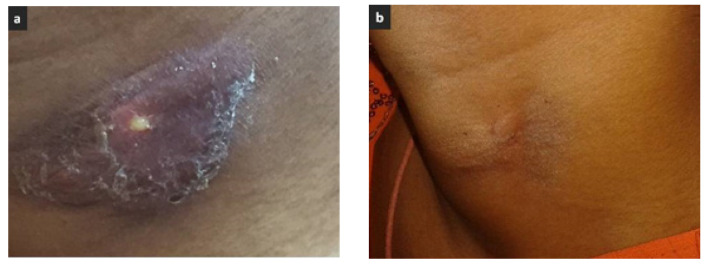
Patient with cervical abscess due to melioidosis on (**a**) initial presentation and (**b**) four months after ceftazidime therapy.

A 27-year-old village-dwelling housewife presented with history of mild and continuous fever, cough without expectoration and left-sided neck swelling for one month. The lesion initially started as a small marble-sized tender nodule progressing to a full-fledged painful neck abscess within the next 25 days and started to rupture and discharge spontaneously with oozing of pus (Figure 3a). Examination revealed the swelling to be about 3.0 × 2.0 cm, soft to firm, and tender, with a thick, white discharging pus. The patient was afebrile, had blood pressure 110/70 mmHg, heart rate 86/min, and respiratory rate 20/min. Laboratory investigations showed an extremely low hemoglobin level of 79 g/L and raised ESR of 80 mm/h. Other haematological and biochemical indices, including plasma glucose level, were within normal ranges. The discharging pus was negative for acid fast bacilli (AFB), but Gram stain showed eight to ten pus cells per oil immersion field (oif) with scanty gram-negative bacteria. Culture revealed growth of *B. pseudomallei* sensitive to ceftazidime, co-trimoxazole, meropenem, imipenem and doxycycline; MIC of ceftazidime was 4 µg/mL [13]. The patient was started on standard regimen of parenteral ceftazidime for three weeks followed by oral co-trimoxazole along with oral iron therapy for correction for anemia. At her latest follow-up four months after initial diagnosis of melioidosis, the lesion had healed without any residual clinical sign of infection (Figure 3b).

### 1.3. Case 3

A 30-year-old male, working as an office staff presented with history of swelling and pain in the right pre-auricular region of 20 days duration. He had difficulty in opening his mouth and also complained of fever since the last 10 days. Examination revealed a cystic, warm, fluctuant swelling approximately 30 × 20 mm in diameter, with induration and tenderness. Laboratory investigations showed hemoglobin level 151 g/L, total leucocyte count (TLC) 15.8 × 10^9^/L; (55% polymorphs, 32% lymphocytes, 8% eosinophils, 5% monocytes), raised ESR 40 mm/h and random blood sugar level of 73 mg/dL. Non-contrast computed tomography (NCCT) revealed an irregular hypodense lesion of 43 × 22 × 23 mm diagnosed as a right parotid abscess. Approximately 5 mL of seropurulent aspirated fluid from the abscess received in the Microbiology Laboratory yielded a pure growth of *B. pseudomallei*. A direct Gram stain examination of the sample revealed more than 100 pus cells per oif with moderate number of gram-negative rods. The isolate was resistant to ceftazidime and amoxycillin-clavulanate, but susceptible to co-trimoxazole, imipenem, meropenem and doxycycline. In view of ceftazidime resistance, the patient was treated with parenteral meropenem for three weeks with advice of oral co-trimoxazole for six months along with incision and drainage of the abscess. Complete healing of the lesion was noted at a five-month follow-up period.

### 1.4. Case 4

A 17-year-old female student was admitted in view of a progressively increasing right pre-auricular swelling since the last 7 days associated with pain and fever. She was found to be a resident of the same village as Case 3. Laboratory investigations showed hemoglobin level 115 g/L, TLC 31.1 × 10^9^/L (85% polymorphs, 10% lymphocytes, 3% eosinophils, 2% monocytes), raised ESR 60 mm/h and random blood sugar level of 86 mg/dL. NCCT revealed a heterogenous hypodense lesion of 63 × 32 × 42 mm diagnosed as a right parotid abscess. Incision and drainage was performed and approximately 20 mL of frank pus drained which showed 40–50 pus cells per oif with plenty of gram-negative bacilli and yielded *B. pseudomallei* on culture. The patient was treated with the standard regimen of parenteral ceftazidime and upon discharge was advised to continue oral co-trimoxazole for a period of six months.

### 1.5. Case 5

A 31-year-old female, presented with a warm, fluctuant left neck swelling for the last 15 days associated with pain and fever. Laboratory investigations revealed TLC of 20.6 × 10^9^/L, raised ESR of 50 mm/h and random blood sugar level of 327 mg/dL. Gram stain examination of a pus aspirate from the abscess showed plenty (>100) of pus cells per oif and culture yielded pure growth of *B. pseudomallei* resistant to amoxycillin-clavulanate. Her lesion healed following incision-drainage and standard therapy for melioidosis with ceftazidime and co-trimoxazole.

### 1.6. Case 6

A five-year-old child, presented with a neck mass of one-month duration, that was gradual in onset, progressively increasing in size and was associated with non-productive cough and evening rise of temperature. She had no history of contact with tuberculosis and was immunized as per age. On examination, her vital signs were stable with a pulse rate of 96/min, a regular respiratory rate of 25/min and a temperature of 37 °C. Weight and height were within normal ranges. On local examination, there was a single cervical mass with multiple enlarged lymph nodes, which were firm, tender, matted, mobile and not fixed to the overlying skin. There was no erythema or discharge. Laboratory investigations showed TLC 14.4 × 10^9^/L (66% polymorphs, 30% lymphocytes, 3% eosinophils, 1% monocytes), haemoglobin 126 g/L, and ESR 45 mm/h. A biopsied pus sample on Gram stain showed 50–60 pus cells per oil immersion field with gram-negative bacteria. Culture revealed growth of *B. pseudomallei* sensitive to standard antibiotics. Along with incision and drainage, the child was treated with IV ceftazidime following which she was discharged with instructions to parents to complete an oral course of co-trimoxazole.

### 1.7. Case 7

A 35-year-old female had a swelling on left side of neck (approximately 60 mm × 50 mm in size, soft in consistency, erythematous and tender) for the last three months associated with evening rise of temperature. Following growth of *B. pseudomallei* in culture of the drained pus, the patient was started on standard therapy for meliodiosis and discharged.

### 1.8. Case 8

An 18-year-old male presented with right neck abscess since the last 15 days associated with pain and fever. *B. pseudomallei* grown from the abscess pus showing 70–80 pus cells per oif with plenty of gram-negative bacilli was susceptible to ceftazidime, co-trimoxazole, imipenem, meropenem and doxycycline but resistant to amoxicillin-clavulanate. The lesion was completely healed after incision and drainage and therapy with ceftazidime.

### 1.9. Case 9

A 53-year-old male, farmer by occupation, was admitted with history of fever and a spontaneously developing painful swelling on right scalp of one-month duration. The patient was a known diabetic and the random blood sugar level on presentation was 364 mg/dL. Other laboratory investigations revealed TLC of 16.9 × 10^9^/L and raised ESR of 60 mm/h. A pus culture which on Gram stain showed plenty of pus cells and gram- negative rods grew *B. pseudomallei* in pure culture. Incision and drainage was done and the patient commenced on parenteral ceftazidime for three weeks. He was advised a 6-month course of oral co-trimoxazole upon discharge.

## 2. Discussion

Neck mass due to melioidosis can present either as suppurative infection (acute or chronic) or as relapse [14]. Suppurative infections due to melioidosis presenting as neck mass or abscesses of the head and neck region have been described from various endemic regions of the world, like Malaysia [5,6,7], Indonesia [8], Taiwan [9] and India [10,11,12]. In non-endemic regions like the USA or UK, melioidotic neck masses have usually been described either in returning travelers [15] or in non-indigenous immigrant populations [14]. Diagnostic confusion with tuberculosis can occur, especially as the ethnic groups with a high incidence of both diseases are similar [16]. Incidentally, co-infection of *B. pseudomallei* with *Mycobacterium tuberculosis* has also been described on few occasions [17,18].

The present series describes nine patients diagnosed as head and neck melioidosis in a tertiary referral hospital in Eastern India over a period of two and half years, based on the isolation of *B. pseudomallei* from the discharging or aspirated pus specimen obtained from the lesions of the patients. Of these, six (66.7%) were female and only one (11.1%) was a child. The age range varied from 5–53 years. By occupation, the majority (4, 44.4%) were housewives, three (33.3%) were students and one each (11.1%) was a farmer and officegoer. This shows the ubiquitous presence of the organism in soil and water and susceptibility of all occupational groups to the disease. Majority (7/9, 77.8%) cases occurred during the rainy season (May to September) and were from the rural area (6/9, 66.7%) as has been reported previously [3,4]. Since the organism is present in soil and surface water, most melioidosis cases are believed to result from bacterial inoculation or inhalation. Heavy rains precipitate flooding, which facilitates churning of *B. pseudomallei* to the surface soil, aerosolizing the bacteria and increasing the exposure potential during the rainy season. Recent evidence has increasingly suggested ingestion to be an important alternative route of infection, by isolating *B. pseudomallei* from water supply of the melioidosis affected community during outbreaks and also by detecting similar genotypes in water and clinical samples [19,20]. In our series, two cases from same locality of a village presented with parotid swelling within a span of 10 days (Cases 3 & 4). On enquiry, it was found that they shared the drinking water from the same well raising the possibility of contaminated water as the source of infection though the water could not be subjected for bacterial isolation. 

The most common presentation in the current series was submandibular lymphadenitis or abscess (5/9, 55.5%), followed by parotid abscess (2/9, 22.2%), and abscess in the supraclavicular and scalp area (1/9, 11.1% each). In a study on pediatric melioidosis in Pahang, among patients with localized melioidosis, head and neck involvement (5 patients, 83.3%) was the most common presentation (2 patients with cervical abscesses, 1 with submandibular abscesses and 2 with acute suppurative parotitis) [6]. In another report on melioidosis of head and neck described in 4 patients, the cases presented were parotid abscess, acute sinusitis, acute suppurative lymphadenitis and chronic suppurative otitis media [7]. Further, in the present case series, only three (33.3%) patients had an underlying disease, none had associated bacteremia, and all had an uneventful recovery following appropriate treatment. Thus, people without any apparent predisposing factor or any occupational risk can also contract the disease, however, such apparently healthy persons, are likely to manifest *B. pseudomallei* infection more often as a localised disease and are likely to have a favourable outcome if provided with timely intervention (Table 1).

In regard to the laboratory characterization, all isolates of *B. pseudomallei* in the current study was susceptible to meropenem, trimethoprim-sulfamethoxazole, and doxycycline, while 1 (11.1%) and 4 (44.4%) isolates were resistant to ceftazidime and amoxicillin-clavulanic acid, respectively. The patient whose isolate was resistant to ceftazidime was successfully treated with meropenem. Overt resistance to both ceftazidime and amoxicillin-clavulanic acid in clinical *B. pseudomallei* isolates as well as variations in ceftazidime and amoxicillin-clavulanate susceptibilities within a clonal infection has been reported in a few instances before [21,22]. Carbapenems are considered as alternative drugs because of their active in vitro activity, post-antibiotic effect and association with decreased endotoxin release [21,22].

## 3. Conclusions

Melioidosis is an emerging entity in India. Prognosis is excellent with early detection and appropriate management, especially of localized infections like abscesses of the head and neck area. Since melioidotic abscesses in the cervicofacial and head and neck region are likely to be confused with cold abscesses caused by *M. tuberculosis* in a tuberculosis- endemic country like India, an increased vigilance is necessary because of the widely divergent treatment modalities of the two disease entities. It has been estimated that melioidosis is severely under-reported in many countries of Southeast and South Asia, including India, and could be much more prevalent than what is available in the literature [2]. This may be because of severe underreporting due to a low clinical index of suspicion or misidentification of the organism as culture contaminants or as *Pseudomonas* species. We suggest that melioidosis should be considered in the differential diagnosis of abscesses in the head and neck area in patients from endemic areas, particularly from rural settings. Currently an under-recognized entity, this report highlights the role of *B. pseudomallei* as an emerging cause of head and neck abscess and emphasizes the increasing need of awareness for melioidosis among clinicians and microbiologists.

## Figures and Tables

**Table 1:** Clinical and bacteriological summaries of nine patients with melioidosis of head and neck.

Features	Case 1	Case 2	Case 3	Case 4	Case 5	Case 6	Case 7	Case 8	Case 9
Age (in years)	49	27	30	17	31	5	35	18	53
Sex	F	F	M	F	F	F	F	M	M
Occupation	Housewife	Housewife	Office-staff	Student	Housewife	Student	Housewife	Student	Farmer
Month of presentation	July 2015	September 2015	August 2016	September 2016	January 2017	January 2017	May 2017	June 2017	September 2017
Area	Semi-urban	Rural	Rural	Rural	Rural	Urban	Rural	Rural	Semi-urban
Type of abscess	Cervical (submandibular lymphadenitis)	Parotid and cervical (sub-mandibular abscess)	Parotid	Parotid	Cervical (submandibular lymphadenitis)	Cervical (submandibular lymphadenitis)	Cervical (abscess in the supraclavicular area)	Cervical (submandibular lymphadenitis)	Scalp abscess
Site of abscess	Left	Left	Right	Right	Left	Right	Left	Right	Right
Size of abscess in CT (in mm)	48.5 × 49.5 × 56.9	34 × 20 × 28	43 × 22 × 23	63 × 32 × 42	33 × 22 × 26	ND	61 × 44 × 53	58 × 47 × 49	ND
Duration of symptoms	20 days	1½ months	20 days	7 days	15 days	1 month	3 months	15 days	20 days
Underlying disease	DM	-	-	-	DM	-	-	-	DM
TLC	13.9 × 10^9^/L	9.5 × 10^9^/L	15.8 × 10^9^/L	31.1 × 10^9^/L	20.6 × 10^9^/L	14.4 × 10^9^/L	9.4 × 10^9^/L	17.5 × 10^9^/L	16.9 × 10^9^/L
ESR (mm at first hour)	60	80	40	60	50	45	65	40	60
HIV serology	NR	NR	NR	NR	NR	NR	NR	NR	NR
Antibiotic susceptibility pattern -									
CAZ	S	S	R	S	S	S	S	S	S
MEM/IPM	S	S	S	S	S	S	S	S	S
TS	S	S	S	S	S	S	S	S	S
AMC	S	S	R	S	R	S	R	R	S
DO	S	S	S	S	S	S	S	S	S
Surgical management	Incision and drainage	Incision and drainage	Incision and drainage	Incision and drainage	Incision and drainage	Incision and drainage	Incision and drainage	Incision and drainage	Incision and drainage
Intensive therapy	IV CAZ × 3 weeks	IV CAZ × 3 weeks	IV MEM × 3 weeks	IV CAZ × 3 weeks	IV CAZ × 3 weeks	IV CAZ × 3 weeks	IV CAZ × 3 weeks	IV CAZ × 3 weeks	IV CAZ × 3 weeks
Maintenance therapy	Oral TS × 6 months	Oral TS × 6 months	Oral TS × 6 months	Oral TS × 6 months	Oral TS × 6 months	Oral TS × 6 months	Oral TS × 6 months	Oral TS × 6 months	Oral TS × 6 months
Outcome (2–6 months follow-up)	Lesion healed	Lesion healed	Lesion healed	Lesion healed	Lesion healed	Lesion healed	Lesion healed	Lesion healed	Lesion healed

F—Female; M—Male; CT—Computed tomography; ND—Not done; DM—Diabetes mellitus; CAZ—Ceftazidime; MEM—Meropenem; IPM—Imipenem; TS—Trimethoprim-sulfamethoxazole; AMC—Amoxicillin-clavulanic acid; DO—Doxycycline; S—Sensitive; R—Resistant; IV—Intravenous; HIV—Human immunodeficiency virus; HBsAg—Hepatitis B surface antigen; NR—Non-reactive; TLC—Total leucocyte count; ESR—Erythrocyte sedimentation rate.
